# Synchronization of Dairy Cows Does Not Limit the Behavioral Response to Treatment in Mixed Treatment Experimental Designs

**DOI:** 10.3389/fvets.2016.00098

**Published:** 2016-11-11

**Authors:** Meagan T. M. King, Robin E. Crossley, Trevor J. DeVries

**Affiliations:** ^1^Department of Animal Biosciences, University of Guelph, Guelph, ON, Canada

**Keywords:** experimental design, feeding behavior, dairy cow, synchronization, facilitation

## Abstract

In many research studies, animals assigned to different treatments are housed adjacently or together in a group. One critique of these designs has been the potential role of behavioral facilitation and synchronization between animals on different treatments in mixed treatment groups (heterogeneous groups). To evaluate this, we compared the synchrony of feeding behavior between dairy cows housed in heterogeneous groups to cows exposed to the same treatment simultaneously (homogenous groups). Twenty-four cows were exposed to each of the two treatments over 21 days in a replicated cross-over design. Treatments were two different schedules of timing of feed delivery: (A) feeding at milking time and (B) feeding halfway between milking times. For the last 7 days of each treatment period, feeding behavior was recorded electronically. Kappa coefficients were calculated for each animal within each group, as an estimate of agreement that any two cows within a group (i.e., each individual and each other cow in her group) would both be engaged in feeding activity for any hour of the day. The level of synchrony was similar for cows within homogenous groups (kappa = 0.31 ± 0.030) compared with cows on the same treatment within heterogeneous groups (kappa = 0.32 ± 0.037). Within heterogeneous groups, cows on the same treatment were nearly 50% more synchronized with each other than with those on the other treatment (kappa = 0.22 ± 0.029). These results suggest that synchronization of feeding behavior does not restrict our ability to impose different treatments on individual cows within a group.

## Introduction

Cattle are gregarious animals that synchronize their behavioral patterns through social facilitation. Facilitation of feeding behavior has been shown to play a role in learning in both dairy and beef cattle. For example, heifers learn to graze more quickly when on pasture with experienced cows (1), and they can learn feed location from other heifers in experimental mazes (2). Feeding synchrony may be influenced by endogenous and exogenous factors. Exogenous cues for highly synchronized feeding activity include management events such as fresh feed delivery and return from milking (3, 4), while the milking process itself has a continuous influence on cow behavior and synchrony [i.e., routine group milkings in conventional parlors compared with unscheduled individual milkings in automated milking systems which may reduce the synchrony of feeding and lying behavior (5, 6)]. Type of housing system can also affect synchrony of feeding and lying behavior of cows [i.e., free-stalls vs. straw yards (7, 8)]. Finally, group size and stocking density can influence synchrony. Livestock, such as dairy cows and heifers (9–11), sheep (12, 13) and poultry (14), housed in smaller groups or at lower stocking densities display more behavioral synchrony than when housed in large groups or at higher stocking densities.

While synchrony is a fundamental component of cattle behavior, it also has the potential to be problematic for research, particularly that which focuses on behavioral outcomes. Researchers often house cows on different treatments adjacent to each other (e.g., in tie-stall facilities) or individually assign cows to different treatments within a single group (e.g., in free-stall groups). In these situations, it is possible that the behavior of one animal, within a group or housed adjacently, can influence the behavior of those on the other treatment, especially when treatments are intended to stimulate different behavioral responses throughout the day (15). This could minimize the predicted behavioral response to treatments or increase variability in response, making it more difficult to detect differences between treatments and thus reduce the efficacy of such experimental designs. Ideally, researchers should impose both treatments simultaneously to eliminate confounding variables (e.g., time and location), but this also creates the possibility for social facilitation between animals on different treatments, whether between individual cows within a group or those housed adjacently.

Therefore, the objective of this study was to determine if behavioral synchronization modifies the behavioral response of cows exposed to different treatments within a group. Two different feeding schedules (treatments) were tested in two experimental designs: (1) heterogeneous groups, where cows on different treatments were housed together within a group, and (2) homogenous groups, where all cows in a group were exposed to the same treatment simultaneously. We hypothesized that cows housed in homogenous groups would display more synchrony and stronger behavioral responses, to their respective treatments, compared with cows housed in heterogeneous groups on the same treatment. Furthermore, it was expected that social facilitation would promote similar synchrony between cows within heterogeneous groups, regardless of treatment.

## Animals, Materials, and Methods

We observed 24 lactating Holstein dairy cows, including 8 primiparous (PP) and 16 multiparous individuals (MP; parity = 2.7 ± 1.0; mean ± SD). Cows were 63 ± 25 days in milk, producing 45.9 ± 6.9 kg/day, and weighed 664.3 ± 64.1 kg at enrollment into the study. Four groups of cows were housed six at a time in a free-stall research pen at the University of Guelph, Kemptville Campus Dairy Education and Innovation Center (Kemptville, ON, Canada). Cows were milked three times per day (at 14:00, 21:00, and 07:00 hours) using an automated milking system (Lely A3 Next, Lely Industries N.V., Maassluis, The Netherlands) by moving cows from the research pen into a small holding area adjacent to the milking system. Cows were milked individually and sequentially, while receiving no supplemental feed, and were returned to their pen individually following milking. Animal use complied with the Canadian Council on Animal Care (CCAC) guidelines (16) and was approved by the Animal Care Committee at the University of Guelph (Animal Use Protocol #1640).

**Figure 1:**
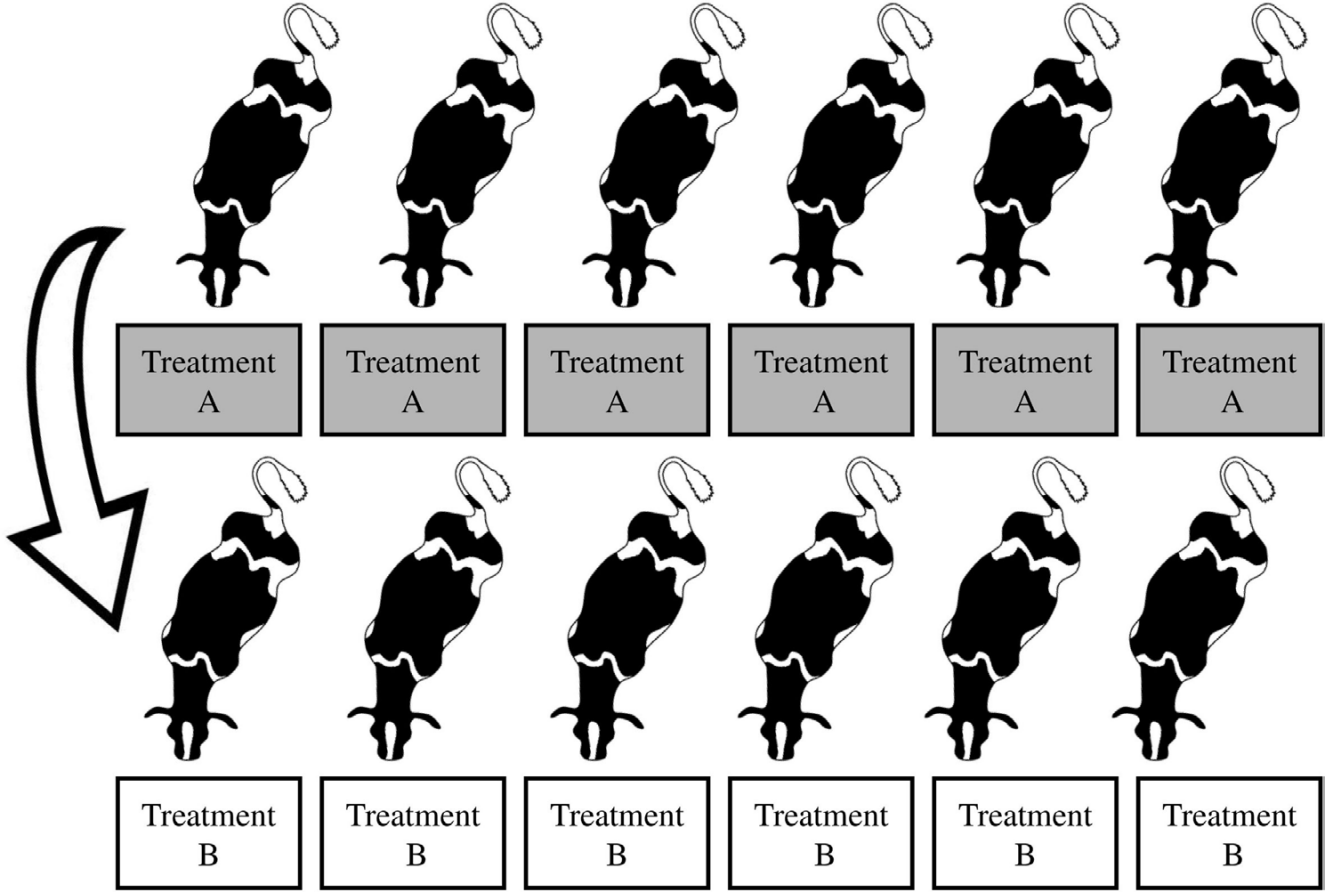
**Homogenous groups and the order of treatment exposure (A and B) for the first group of cows; the second group underwent the reverse order of treatment exposure (B and A)**. Treatment A: cows fed at milking time. Treatment B: cows fed between milkings. Figure credit for cow silhouettes: Jason C. Fisher, Integration and Application Network, University of Maryland Center for Environmental Science (http://ian.umces.edu/imagelibrary/).

**Figure 2:**
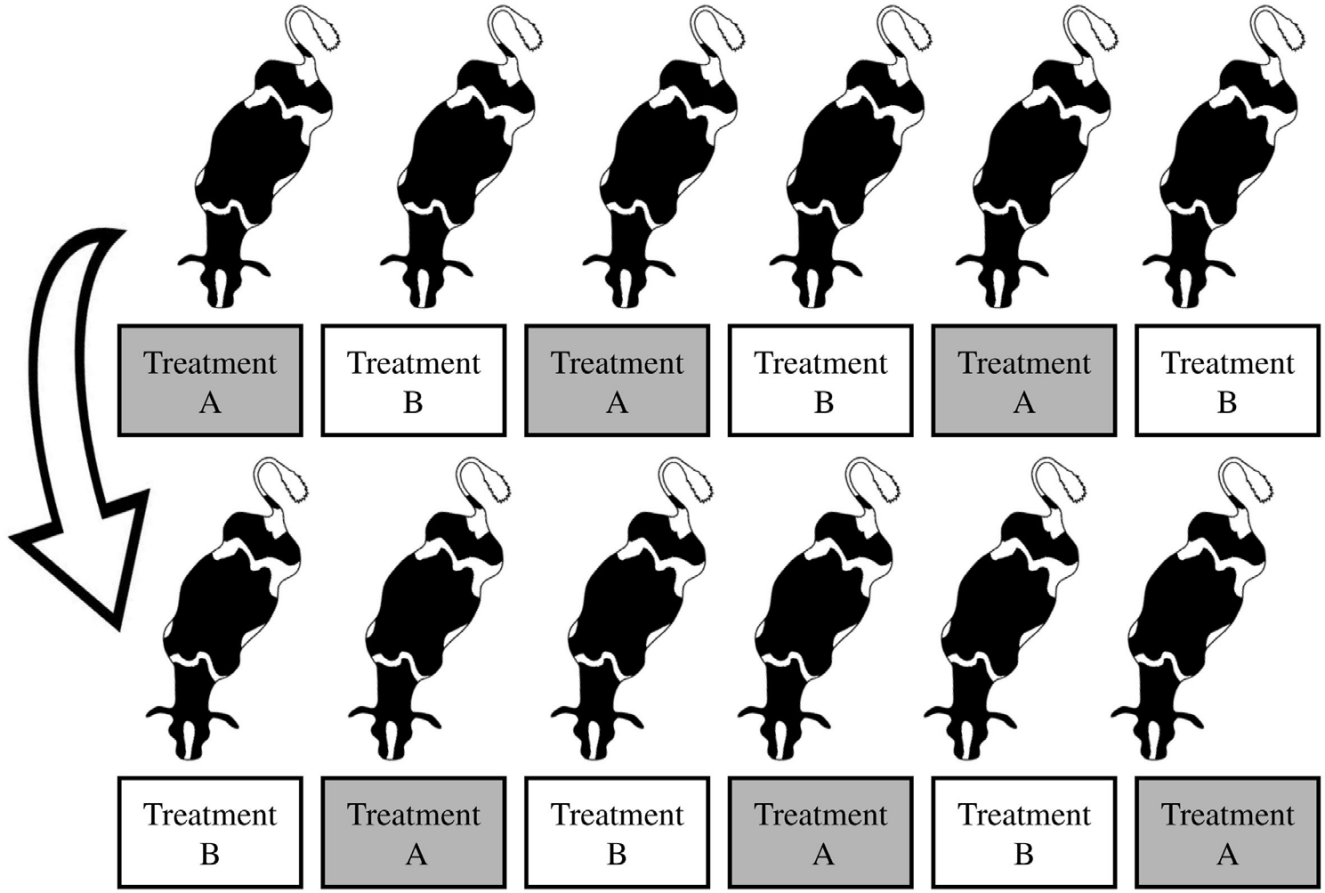
**Heterogeneous groups and the order of exposure for the first group of cows (alternately assigned to each treatment, either A or B); the second group underwent the reverse order of treatment exposure (now B or A)**. Treatment A: cows fed at milking time. Treatment B: cows fed between milkings. Figure credit for cow silhouettes: Jason C. Fisher, Integration and Application Network, University of Maryland Center for Environmental Science (http://ian.umces.edu/imagelibrary/).

Each group of six cows was balanced according to parity, days in milk, and milk production. Within each group, cows were individually assigned and exposed to each of the two treatments in a replicated cross-over design (with groups replicated over time). The first two groups of cows were assigned to the same treatment within each period (homogenous groups; Figure 1). The following two groups were heterogeneous, where cows were alternately assigned to treatments within each period (Figure 2). The order of treatment exposure was reversed for the second replicate of each respective design. Thus, across all 24 cows, the order of treatment exposure was balanced.

Treatments were the manipulation of timing of fresh feed delivery, two times per day, in relation to milking time: (A) feed delivery at milking time (at 14:00 and 07:00 hours) and (B) feed delivery with delay, halfway between milking times (at 17:30 and 10:30 hours, with a 3.5-h delay from milking time). The treatments were selected for a related study, which evaluated the effect of timing of feed delivery on cow behavior and productivity using data from the two heterogeneous groups (4). Cows were exposed to each treatment for 21 days, consisting of 14 days of adaptation to the feeding schedule, followed by a 7-day data collection period of their feeding behavior.

Cows were trained to feed at individually assigned feed bins (Insentec RIC, Marknesse, The Netherlands); this system was previously validated to electronically record every visit to each feed bin (17). Feed was available throughout the entire day, as each cow was fed according to her own intake level, and this amount was adjusted daily for 10% refusals. To test synchrony of behavior within groups, kappa coefficients were calculated for each animal within each group (18). This value provided an estimate of the probability of agreement that two cows within a group (i.e., each individual and each other cow in her group) would both be engaged in feeding activity for any hour of the day. Therefore, for each day, a kappa coefficient was calculated for all combinations of individual cows feeding together within a group. As treatment was applied at the cow level, the experimental unit was cow (4); however, for the purposes of this analysis, to avoid double-counting pairs of observations, the observational unit in our analysis was the pair of cows (*n* = 15 per group). The average of all kappa coefficients for each pair for each day were then averaged across the 7 days of each treatment period and analyzed using the MIXED procedure of SAS 9.4 (SAS Institute Inc., Cary, NC, USA). The model included the fixed effects of experimental design (heterogeneous vs. homogenous) and treatment (i.e., feeding schedule), as well as their interaction. Random effects were group within experimental design, and pair of cows within group and experimental design. Using the CONTRAST statement of SAS, orthogonal contrasts were used to compare the kappa coefficients for cows on the same treatment within heterogeneous groups against cows on the other treatment in heterogeneous groups, as well as against cows on the same treatment in homogenous groups.

## Results and Discussion

There was no difference (*P* = 0.95) in the level of feeding synchrony between cows on the same treatment within a homogenous group (kappa = 0.31 ± 0.030) compared with those on the same treatment within a heterogeneous group (kappa = 0.32 ± 0.037). The latter level of synchrony (between cows on the same treatment in heterogeneous groups) was nearly 50% greater (*P* = 0.0016) than the synchrony between cows on different treatments within heterogeneous groups (kappa = 0.22 ± 0.029). This means that cows were nearly 50% more synchronized with others on the same treatment, regardless of experimental design, than with cows on the other treatment. In contrast to our hypotheses, these results demonstrate that the level of synchronization between cows on the same treatment was similar, whether in homogenous or heterogeneous group. While there was some synchrony between cows on different treatments in heterogeneous groups, the level of synchrony between cows on the same treatment was much higher. These differences in feeding synchrony suggest that behavioral responses associated with response to a specific treatment (i.e., in this case, feed delivery) are driven by the treatment stimulus more so than by the behavior of neighboring cows on another treatment.

These results provide empirical support that heterogeneous treatment experimental designs do not necessarily alter feeding behavior responses to different treatments. The possibility that social facilitation could influence feeding patterns and limit the ability of heterogeneous treatment groups to stimulate behavioral responses was previously proposed (15). With the results of the current study and the known advantage of heterogeneous treatment experimental designs, whereby cows are exposed to both treatments under identical environmental conditions (15), it is appropriate to continue using heterogeneous experimental designs in future studies with predicted behavioral outcomes. Alternatively, homogenous treatment groups (where treatment is assigned at the cow level) should not be utilized because they potentially confound treatment with time and/or location and create more opportunity for systematic error. In order to conduct this experiment however, it was necessary to confound our “treatment” (in this case, the experimental design, not the feeding schedule) with both time and location to collect individual feeding data at available feed bins. The authors recognize this possible limitation; however, the study was designed to assess the relative efficacy of experimental designs that either include confounds or potential behavioral facilitation. Since it was demonstrated that similar behavioral responses to different feeding schedules were elicited regardless of experimental design, the lack of synchrony suggests that heterogeneous groups were neither constrained by experimental confounds nor behavioral facilitation.

It is interesting to note that the current study’s level of synchrony in homogenous groups was greater than the feeding synchrony of cows in another study [kappa = 0.19 (11)] that were also housed in homogenous treatment groups at the same stocking density as the current study. This suggests a high degree of synchrony in the current study, where cows had equal opportunity to access individually assigned feed bins. Alternatively, Winckler et al. (11) fed cows at a post-and-rail feed bunk, which provided cows the opportunity to displace one another and to feed at any location along the bunk. Headlocks and partitions between feed stalls have been demonstrated to drastically reduce the number of aggressive displacements (19, 20). In the current study, the feed bins were more comparable to individual feed stalls or headlocks than to post-and-rail bunks. Since displacement frequency and feeding synchrony are inversely related (11), it is likely that our individual feed bins were the main source of enhanced synchrony. Moreover, cows in the current study had no access to feed in any bin but their own and therefore should have had much less motivation to displace one another.

This study obtained similar kappa coefficients (0.2–0.3) for behavioral synchrony compared with studies with other livestock species. The most applicable comparison is to grazing sheep studied by Rook and Penning (21), with kappa coefficients of approximately 0.1 for feeding behavior. Perhaps those values were lower because animals outdoors likely maintain a higher level of vigilance in order to detect predators, whereas the cows in the current study were housed indoors without this threat. A less direct comparison would be a study of pigs, at various ages, that increased their synchronization of object-directed behavior with age while becoming less synchronized in terms of states of activity and inactivity (22). The youngest group (sucklers) in that study was not fed milk *ad libitum*, whereas the older groups (weaners and growers) had feed available throughout the day (similar to cows in the current study). Therefore, younger pigs’ bouts of feeding behavior and inactivity were more synchronized and declined over time. Kappa coefficients observed in that study were approximately 0.9 for inactivity, 0.7–0.9 for activity, and ranged from 0.1 to 0.5 for object-directed behavior.

The small group sizes (six cows per group) utilized in the current study may have allowed for greater synchrony, since previous work has shown synchrony to decrease with group size (10, 13, 14). Group size was held constant in this study because our objective was to compare homogenous and heterogeneous treatment groups, and furthermore, the research facility available dictated these group sizes. Additionally, it was not our goal to examine feeding behavior under commercial free-stall conditions with competition at a feed bunk. These results are, therefore, applicable to experimental situations where cows are assigned to treatment within a group, without competition to access their feed.

## Conclusion

In summary, the results of this analysis suggest that the level of feeding synchrony between a cow and those on a different treatment in the same group was much lower than the level of synchronization between cows on the same treatment. These results suggest that synchronization of feeding behavior does not restrict our ability to impose different treatments to individual cows within a group. Thus, this provides empirical support for researchers to continue utilizing heterogeneous designs, where treatment can be applied at the cow level, to eliminate confounding variables and maximize the efficacy of dairy cow studies with behavioral outcomes.

## Author Contributions

MK designed and conducted the study, collected data, and wrote this manuscript for submission. RC also conducted the study, collected data, and reviewed this manuscript for submission. TD oversaw and designed the study, analyzed the data, and reviewed this manuscript for submission.

## Conflict of Interest Statement

The authors declare that the research was conducted in the absence of any commercial or financial relationships that could be construed as a potential conflict of interest.
